# Examining the Human Activity-Intensity Change at Different Stages of the COVID-19 Pandemic across Chinese Working, Residential and Entertainment Areas

**DOI:** 10.3390/ijerph20010390

**Published:** 2022-12-26

**Authors:** Shuang Ma, Kang Cao, Shuangjin Li, Yaozhi Luo, Ke Wang, Wei Liu, Guohui Sun

**Affiliations:** 1 College of Civil Engineering and Architecture, Zhejiang University, Hangzhou 310058, China; 2 Graduate School of Advanced Science and Engineering, Hiroshima University, Higashihiroshima 739-8529, Japan; 3 Institute for Health and Environment, Chongqing University of Science and Technology, Chongqing 401331, China; 4 Beijing Key Laboratory of Environment and Viral Oncology, Faculty of Environment and Life, Beijing University of Technology, Beijing 100124, China

**Keywords:** COVID-19, location-based services (LBS) data, nationwide, multi stages

## Abstract

The COVID-19 pandemic has already resulted in more than 6 million deaths worldwide as of December 2022. The COVID-19 has also been greatly affecting the activity of the human population in China and the world. It remains unclear how the human activity-intensity changes have been affected by the COVID-19 spread in China at its different stages along with the lockdown and relaxation policies. We used four days of Location-based services data from Tencent across China to capture the real-time changes in human activity intensity in three stages of COVID-19—namely, during the lockdown, at the first stage of work resuming and at the stage of total work resuming—and observed the changes in different land use categories. We applied the mean decrease Gini (MDG) approach in random forest to examine how these changes are influenced by land attributes, relying on the CART algorithm in Python. This approach was also compared with Geographically Weighted Regression (GWR). Our analysis revealed that the human activity intensity decreased by 22–35%, 9–16% and 6–15%, respectively, in relation to the normal conditions before the spread of COVID-19 during the three periods. The human activity intensity associated with commercial sites, sports facilities/gyms and tourism experienced the relatively largest contraction during the lockdown. During the relaxations of restrictions, government institutions showed a 13.89% rise in intensity at the first stage of work resuming, which was the highest rate among all the working sectors. Furthermore, the GDP and road junction density were more influenced by the change in human activity intensity for all land use categories. The bus stop density was importantly associated with mixed-use land recovery during the relaxing stages, while the coefficient of density of population in entertainment land were relatively higher at these two stages. This study aims to provide additional support to investigate the human activity changes due to the spread of COVID-19 at different stages across different sectors.

## 1. Introduction

The rapid spread of COVID-19 has caused more than 600 million infections and 6 million deaths worldwide as of December 2022 (available at: https://covid19.who.int/, accessed on 1 December 2022). To cope with the COVID-19 outbreak, a number of cities implemented shutdown policies to restrict human outdoor activity, work and social interaction. In China, the lockdown of cities commenced on 23 January 2020. To prevent further dissemination, the central government announced the highest state of emergency (level-1) on 29 January 2020, and by 30 January 2020, cities in the whole of the country were in lockdown. All residents were required to reduce their inter-city travel and intra-city activities due to the strict policies. This strict lockdown was efficient in reducing COVID-19 transmission in China [1,2] (According to the National Health Commission, until 9 February 2020, new infection cases nationwide (except in the Hubei Province) had continued to decline for 6 days and the number of newly confirmed cases in the first-tier cities including Beijing, Shanghai, Guangzhou and Shenzhen was 27). Apart from China, numerous countries around the world also proposed mobility restriction strategies. By April 2020, over 90 countries had been locked down with more than half of humanity being asked to stay at home. The effectiveness was checked by several scholars, for instance, Sharma et al. checked the reduction of death from COVID-19 through human mobility restrictions such as the closing of educational institutions in seven European countries (Austria, the Czech Republic, England, Germany, Italy, the Netherlands, and Switzerland) [3]. In China, 10 February 2020, marked the first working day for the majority of businesses in China. The Spring Festival holiday, originally scheduled to end on 30 January, was extended to control the spread of the virus. On that day (work resuming at the initial stage), more than 7000 among the 22,000 registered enterprises in China’s high-tech hub, Zhongguancun, resumed work [4] and approximately 70 per cent, over 5000, of the Shunde-based workforce returned to work [5]. By 18 February (almost total work resuming), most enterprises and government institutes had resumed stable working. According to the press conference held by the Joint Prevention and Control Mechanism of the State Council on 19 February, the resumption rate in large-scale enterprises in the major economic provinces (cities) exceeded 50%. From this background, it is clear that the human activity intensity was strongly affected by COVID-19 in the lockdown and relaxation stages.

Capturing human activity mobility is particularly valuable during the spread of COVID-19. Large technical companies, such as Google, have published human mobility reports. The availability and reliability of multi-source big data has been recognized as an added opportunity to capture the real-time trends in mobility change across the world during the novel coronavirus period [6,7,8]. For instance, mobile phone data was used by Oliver et al. (2020) to quantify the promotion of social distancing [8]. Similarly, Gao et al. (2020) applied mobile phone-location data to reveal the effects of, and geographic variation in, social distancing on the spread of COVID-19 [9]. Unfortunately, as far as we know, no published Smartphone data was available nationwide in China during COVID-19. Airlines have also been widely used by scholars, such as Kraemer et al. (2020), to capture inter-city mobility connections during COVID-19 [10], but there remains a lack of sufficient information regarding intra-city connections. Location-based services (LBS) data has also been employed to quantify mobility variations [11]. For instance, Chinazzi et al. (2020) used Baidu LBS data to determine the mobility variations in Mainland China during COVID-19 [1]. Ma and Long (2019) applied Tencent LBS data to evaluate the human activity intensity in wilderness delineation [12]. However, although the LBS data records the location records of users, it does not record the mobility traces of the users. In our study, one human activity represents one location request from the Tencent-related Apps, which records a geographical position. The data has a 5 km × 5 km resolation, thus the human activity intensity refers to the total count for each grid cell in a selected time period.

The spread of COVID-19, as well as other communicable diseases, is due to transmission from person to person [13]. The human activity intensity is imperative, as while it cannot directly reflect the face-to-face communication through mobility traces, it does reflect possible social contacts. It is obvious that the denser the social contacts, the greater risk there is to be infected by COVID-19. Capturing the human activity intensity serves as a base for evaluation, or intervention, during the spread [8]. It is also important for city management and planning, as well as for future public health decision-making [14]. On the one hand, human activity-intensity changes can be used to evaluate the impact of interventions, such as a lockdown to prevent the spread of COVID-19. On the other hand, it can be used to evaluate the effects of the relaxation strategies, such as work resuming, in different countries. In addition, despite some work and entertainment sectors resuming, it may be difficult for the human activity intensity to return to the high level it was prior to the COVID-19 outbreak. Thus, knowledge of the human activity change intensity can help to examine the impact of the lockdowns and relaxation strategies across different business sectors and would also be beneficial for financial subsidies or support. Nevertheless, there are few publications specifically focusing on the human activity-intensity change at different stages of the COVID-19 pandemic.

In aiming to fill the current research gaps, the first objective of this study was to explore the human activity-intensity change at three different stages (the lockdown of cities, work resuming at the initial stage and total work resuming) of the COVID-19 pandemic. We assumed that the human activity-intensity change at the different stages of the COVID-19 spread varied across different locations;, thus, the second objective was to evaluate the differences in the activity intensity associated with various work and entertainment sectors. The third objective was to examine the importance of land indicators, including the administrative level, urban and rural use, density of the population, accumulative infection cases, GDP and built environment factors (i.e., built-up areas, bus stop density and junction density), to the change differences. We firstly used nationwide points of interest (POIs) to identify the residential, working, entertainment and mixed-use land, and then used Tencent LBS data on four normal days, i.e., a non-holiday and non-Friday, to evaluate the human activity intensity at different stages. We then evaluated the impact factors for the change differences in the human activity intensity through the mean decrease Gini approach. A significant contribution of this study is to clarify how human activity intensity was influenced by COVID-19 at different stages and whether the changes differed across different work and entertainment sectors. With this knowledge the second contribution of this study is an evaluation of the policy effectiveness for human mobility restrictions and relaxation. This study is also beneficial to support government decision-making in relation to subsidies and financial support.

The structure of this paper is organized as follows. First, we explain the study background and objective, as well as the limitation of current studies. A literature review is then provided to better position this paper in the literature. Third, the data and adopted methods of identifying the land use categories and the MDG approach are briefly explained. Fourth, the changes in human activity intensity at different stages for the different land use functions and the importance of land attributes in the changes are analyzed in detail. Finally, we summarize the findings and limitations of this study.

## 2. Literature Review

Previous studies of human activity during the COVID-19 pandemic have focused on how the human mobility reduction due to COVID-19 has had an impact on three aspects: wildlife [15], air pollution [16,17] and the global climate [18]. For example, Forster et al. (2020) stated that global NO*_x_* emissions had declined by as much as 30% in April compared to the start of the year, contributing to short-term cooling, [19]. Many researchers have also examined the efficiency of compulsory or non-compulsory mobility restrictions for humans in preventing COVID-19 [6,10,20,21]. Kraemer et al. (2020) demonstrated that the spatial distribution of COVID-19 cases in China was explained well by human mobility data. Following the implementation of control measures, this correlation decreased and the growth rates became negative in most locations, according to the population flow data from Baidu Inc. [10]. Engle, Stromme and Zhou (2020) found that a rise in the local infection rate from 0% to 0.003% was associated with a 2.31% reduction in mobility through GPS data in the US [22]. Cartenì, Francesco, and Martino (2020) suggested that, in Italy, mobility habits represent one of the variables that explains the number of COVID-19 infections jointly with the number of tests/day and some environmental variables [23]. Through mobile phone data, Yabe et al. (2020) explained that human mobility behavior decreased by around 50%, which showed a correlation with the decreasing number of COVID-19 cases in Tokyo [24].

A few studies examined the human mobility changes during the COVID-19 pandemic to monitor the impact of a lockdown. For instance, Couture et al. (2020) investigated how pandemic-induced reductions in activity vary across people and places using mobile data in all the US states to produce a location-exposure index and a device-exposure index [25]. Pepe et al. (2020) used 70,000 de-identified smartphone users’ data in Italy to assess the mobility changes in Italy following the national lockdown by creating a dataset from three mobility metrics: the origin-destination movement between Italian provinces, the radius of gyration and the average degree of a spatial proximity network [14]. Furthermore, the impact of government measures, and economic and social factors on the degree of mobility change have been examined by a handful of scholars [26,27,28], with Bonaccorsi (2020) suggesting that the impact on mobility during the lockdown was stronger in municipalities with a higher fiscal capacity [26]. In China, based on the Baidu Migration and China’s national statistical reports, Liu, Luo and Wang (2020) suggested that mobility restrictions reduced the inter-provincial in-migration flow by 63% and the out-migration flow by 62% from late January to early May in 2020, but the effects varied significantly across provinces using Difference-in-Differences model [29].

However, there are a few limitations in the current studies. Firstly, little research has evaluated how activities changed during different stages of the COVID-19 spread, including both mobility restrictions and relaxations. Secondly, the different intensity changes across various work and entertainment sectors have not been examined. In addition, as far as we know, there remains no research evaluating the human activity-intensity change to monitor the relaxation in China and explore the influencing factors.

## 3. Data

### 3.1. Location-Based Services (LBS) Data from Tencent Company

The LBS data is from Tencent Company (available at: http://www.qq.com, accessed on 1 December 2022), one of the most popular social network platforms representing China’s largest databases of online footprints. The LBS data records the location of smart phone users who are using a Tencent application, such as the QQ App, WeChat, Tencent Map, Tencent Weibo and Tencent Browns, and other mobile applications that provide LBS services. In 2017 the Tencent social networking platform had over 60 million shares per day (Ma and Long, 2019 [12]). This nationwide vector data has 5 km × 5 km resolution and the human activity intensity was recorded every half hour at each grid cell on four regular weekdays, i.e., a non-holiday and a non-Friday (Friday was excluded to avoid its impact on entertainment activity intensity on a Friday night), with the dates being 19 November 2019; 4 February 2020; 10 February 2020 and 18 February 2020. This represents the activity intensity before COVID-19 (Chinese health authorities began monitoring infection cases of COVID-19 in Wuhan, China at the beginning of December 2019 (Chinazzi et al. [1]), thus we used the human activity intensity at 19 November to represent the human activity intensity before COVID-19.), during the lockdown of the whole country, at the first stage of work resuming and at total work resuming, respectively.

### 3.2. Points of Interest (POIs) in 2015 in China

The POIs used in this study are collected from the Amap electronic navigation map. The original dataset contains two levels of categories, with 23 categories in the first level and 267 types in the second level representing all the different functions in a city. We used the first level categories of residential community, community service, private company, office building, financial sector, legal sector, government institution, education institution, commercial site, hotel, tourism, sport/gym and catering site based on Li, Long and Dang (2018) and sorted them into “residential activity”, “working activity” and “entertainment activity” to classify the residential lands, working lands, and entertainment lands (Table 1) [30]. We have to clarify some decisions on data preparation. Firstly, the POIs representing transport station names, indoor facilities, incident and events, address and road furniture were not sorted into any “residential activity”, “working activity” or “entertainment activity” class. Secondly, multi-function POIs, such as catering sites and commercial sites in the first level categories, can create many job opportunitiessince their main function is entertainment in a whole country; therefore, they were sorted into “entertainment activity”. Furthermore, we classified the main work sectors and entertainment sectors for the working grid cells and entertainment grid cells, respectively, to examine the impact of COVID-19 on different business sectors. We also used the spatial locations of bus stops in this data to compute the bus stop density in every study grid cell (5 km × 5 km). 

### 3.3. Gross Domestic Product (GDP) Spatial Distribution in China

The GDP spatial distribution in China reflects the detailed spatial distribution of GDP data across China. This raster data comes from the Resource and Environmental Science Data Center of the Chinese Academy of Sciences in 2015 (available at: http://www.resdc.cn/DOI/DOI.aspx?DOIid=33, accessed on 1 December 2017) with a resolution of 1 km^2^. Each 1 km^2^ grid cell represents the total GDP output value within the grid cell range, and the unit is CNY 10,000 /km^2^. The GDP measures both the economy’s total income and the economy’s total expenditure, and determines the economic development level. It is argued to be an important factor resulting in the human mobility differences under similar policies during the COVID-19 spread in Italy [26]. We calculated the GDP within every study grid cell in order to check whether or not the economy caused human activity-intensity changes during the different stages in China in every grid cell.

### 3.4. Spatial Distribution of Population in China

The population distribution dataset is a raster data (resolution at 1 km^2^) reflecting the detailed spatial distribution of the population nationwide from the Resource and Environmental Science Data Center of the Chinese Academy of Sciences (2015) (available at: http://www.resdc.cn/DOI/DOI.aspx?DOIid=32, accessed on 1 December 2017). It is based on the national population statistics by counties, and comprehensively considers the types of land use closely related to the population, the brightness of night-lights and the density of residential areas. The population has direct influences on human activity intensity, but whether or not population difference is an important attribute causing activity-intensity change during a lockdown or relaxation during the pandemic still requires clarification. We used China’s population spatial distribution dataset to calculate the population within every study grid cell. Although other attributes of demography, such as gender and age distributions, are also important, this data is not available at the grid cell level and was not included in this study.

### 3.5. Built-Up Areas in China 

Data regarding the built-up areas in China in 2015 were obtained from the Resource and Environment Data Cloud Platform (available at: http://www.resdc.cn/data.aspx?DATAID=184, accessed on 1 December 2017). This data includes 26 types of land-use classes; we used the built-up area type 51 and the data resolution is 30 m. A built-up area is a physical environment supporting human activity; thus, we also evaluated its relationship with human activity-intensity change. This data was used for calculating the built-up area, which reflects an attribute of built environment in every grid cell. The density of built-up areas has been studied previously for its correlation with the COVID-19 spread [31].

### 3.6. Road Junction Density 

We obtained road network data in China from Amap. The road processing includes three steps according to Long and Liu (2017) [32]. The first step is to use the “merge divided roads” tool in the Generalization toolset in ArcGIS10.2 to merge the road network, thereby merging multiple lanes into a single lane. Second is to use the “thin road network”, also in the Generalization toolset in ArcGIS10.2, to simplify the road network and delete thin side roads. Third is to perform a topology check on the road network and deal with topological errors to ensure there are no broken roads and that there is only one intersection when the roads intersect, using the “must not have dangles”, “must not have pseudo nodes” and “must not intersect” rules in ArcGIS10.2. After the processing we created the road junction through the “feature vertices to points” toolbox in ArcGIS10.2 and then computed the road junction density at every study grid cell. Both the bus stop density collected by POIs mentioned above and the road junction density reflect the transportation connections; thus, they may also have a relationship with human activity-intensity change after restriction or relaxation policies. 

### 3.7. Cumulative Numbers of Confirmed Infection Cases of COVID-19

The spread of COVID-19 is imperative to the human activity intensity. We included the cumulative numbers of confirmed infection cases of COVID-19 at the city level, which were collected by the China University of Geoscience (available at: https://github.com/Estelle0217/COVID-19-Epidemic-Dataset, accessed on 1 December 2022) from the official websites of national, provincial and municipal health organizations. The effects of human mobility restrictions or relaxation strategies can be impacted along with the change of infection cases, especially in countries that have a relatively liberal restrictions policy, such as Japan and the US. The cumulative number of confirmed infection cases is one of the most important factors reflecting the virus condition and therefore, we also selected this as an attribute. 

## 4. Method

### 4.1. Identifying the Land Use Categories in China 

**Figure 1:**
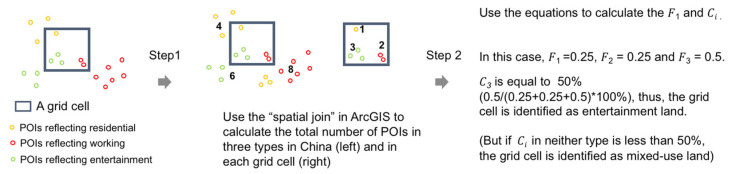
Identifying the land use categories for every grid cell in China.

We firstly computed the total number of POIs reflecting residential, working and entertainment, respectively, in China. This step was conducted in every grid cell using the “spatial join” tool in ArcGIS10.2. Then we used the following equation to identify the land use categories.

(1) $$F_{i}=\frac{n_{i}}{N_{i}}\;\;\;\left(i=1,2,3\right)$$

(2) $$C_{i}=\frac{F_{i}}{\sum_{i=1}^{3}F_{i}}\times 100\%\left(i=1,2,3\right)$$

where $N_{i}$ is the total number of type i in China and $n_{i}$ is the total number of type i in a grid cell. As the total counts of the POIs of “residential” were much fewer than the other two types, the first equation was taken to avoid the influence of the total count differences among the different types on the land use identified. $C_{i}$ is used to evaluate the land use categories; when it reaches 50%, this grid cell will be identified as type i, but, if $C_{i}$ in neither type (residential, working or entertainment) is less than 50%, this means there is no dominant type; therefore, the grid cell is identified as mixed-use land. There were in total 3 types (residential, working and entertainment); therefore, we used i=1,2,3. The processing is shown in Figure 1.

Using a similar method to the above, we further identified the sectors of working and entertainment for the working grid cells and entertainment grid cells, respectively. We firstly computed the total number of POIs reflecting working and entertainment in every sub-category illustrated in Table 1 and then used the above equation to classify the main sectors of the working grid cells and entertainment grid cells. However, this time, type i is a sub-category of working and sub-category of entertainment; i=6 for the working grid cell and i=5 for the entertainment grid. Finally, we divided the work grid cells into private company, office, financial sector, legal sector, government institute and education institute, and divided the entertainment grid cells into commercial sites, hotels, tourism, sports/gyms and catering sites.

### 4.2. Change Rate in Human Activity Intensity 

One human activity refers to one location request from the Tencent Apps through a mobile phone or the Internet, and the human activity intensity relates to the total counts of human activity in a grid cell. As the LBS dataset from Tencent records the human activity intensity at half-hour intervals, in the first step, we calculated the sum of the human activity intensities of four land-use categories (residential, working or entertainment and mixed-use land) on four selected days: 19 November 2019 (LBS day 1); 4 February 2020 (LBS day 2); 10 February 2020 (LBS day 3) and 18 February 2020 (LBS day 4). Moreover, at the same time, when the human activity intensity or number of location requests for a “working” grid cell goes up, the intensity for a “residential” grid cell and an “entertainment” grid cell can go down; thus, we did not use all 48 measurements of human activity intensity in 24 h to sum up the activity intensity. Instead, we selected the main time period of these events to sum up the activity intensity: 22:00–24:00 for residential grid cells, 9:00–17:00 for working grid cells, 17:00–22:00 for entertainment grid cells and 9:00–24:00 for mixed-use grid cells. Then, we defined the change rate as:

(3) $$First\;period=\frac{\left(LBS\;day2\;-\;LBS\;day1\right)}{LBS}day1\times 100\%$$

(4) $$Second\;period=\frac{\left(LBS\;day3\;-\;LBS\;day1\right)}{LBS}day1\times 100\%$$

(5) $$Third\;period=\frac{\left(LBS\;day4-LBS\;day1\right)}{LBS}day1\times 100\%$$

The first period, second period and third period relate to the human activity change during the lockdown, at the first stage of work resuming, and at total work resuming, respectively. One of the study objectives is to evaluate how human activity intensity is influenced by the COVID-19 spread at different stages; thus, in each period, we compared the human activity intensity with the situation before the COVID-19 spread. This also benefits government institutions in providing subsidies or other support for sectors that are not quickly returning to the pre-pandemic human activity intensity.

### 4.3. Evaluating the Influence of Land Attributes to the Change in Human Activity Intensity

There are several methods for exploring the impact factors by time-spatial series data, such as panel data models, spatial econometric models and spatial regression models. They have certain statistical assumptions that generally need to be satisfied. For example, non-strong collinearity among factors is required to ensure minimum variance in the parameter estimation and to guarantee that the estimator is effective [33]. In our study, the relationship between human activity change and its impact factors is a nonlinear relationship and, in addition, in terms of data volume, massive data may cause an overfit by traditional statistic methods. Therefore, we selected the random forest, which is a machine-learning algorithm and can automatically select important variables, as well as flexibly evaluate the complex interaction between variables. Furthermore, this method is insensitive to the multi-collinearity and robustness of massive data [34]. In the random forest approach, we used 55% of the data as the training dataset, 30% as the test dataset and 15% as the validation dataset. We compared the performance of Geographically Weighted Regression (GWR), which can consider the spatial effect of variables on urban recovery, and the random forest model by the Mean Squared Error (MSE) value and adjusted R^2^ in this research according to the Equations (6)–(8) (Table 2). The MSE is useful to evaluate predictions made on the training dataset. A smaller MSE reflects the smaller cost incurred in the prediction. R^2^ is widely used to reflect the goodness of fit line and a higher value of R^2^ means the regression model can better explain the variation of actual values from the mean value. As a result, the performance of the random forest is better than the GWR.

(6) $$MSE=\frac{1}{n}\sum_{i=1}^{n}{\left(y_{i}-{\hat{y}}_{i}\right)}^{2}$$

(7) $$\;R^{2}=1-\frac{\sum {\left(y_{i}-{\hat{y}}_{i}\right)}^{2}}{\sum {\left(y_{i}-\bar{y}\right)}^{2}}$$

(8) $$R_{adj}^{2}=1-\left[\frac{\left(1-R^{2}\right)\left(n-1\right)}{n-k-1}\right]$$

where n is the total number of observations, $y_{i}$ is the ith true data point value, and ${\hat{y}}_{i}$ is the ith estimated data point value, $\bar{y}$ is the mean of data point value, k is the number of independent variables. The lesser the MSE, the bigger the $R_{adj}^{2}$, and the better the regression model is.

The random forest is a model that generates several decision trees that are aggregated to perform a classification task, to select important variables and to calculate the relative importance of each variable [35,36]. Random forest provides mean decrease accuracy (MDA) and MDG methods to measure variable importance [37]. The first method measures the accuracy reduction on out-of-bag samples when the values of the variable are randomly permuted [38]. The MDG is the sum of all decreases in the node impurity due to a given variable (when this variable is used to form a split in the random forest), normalized by the number of trees [39]. The MDG is faster to compute and does not require the use of bootstrap sampling, and it is robust to small perturbations of the data [40]; thus, we apply the MDG to measure the variable importance associated with human activity change in the different periods. We chose the CART algorithm to create regression trees, with the splitting criterion being achievement of each node having a maximum reduction in overall node impurity, where the impurity is measured as the total sum of the squared deviations from the node centers. The computational process was carried out using the “RandomForestRegressor” package from the sklearn module available in Python.

**Figure 2:**
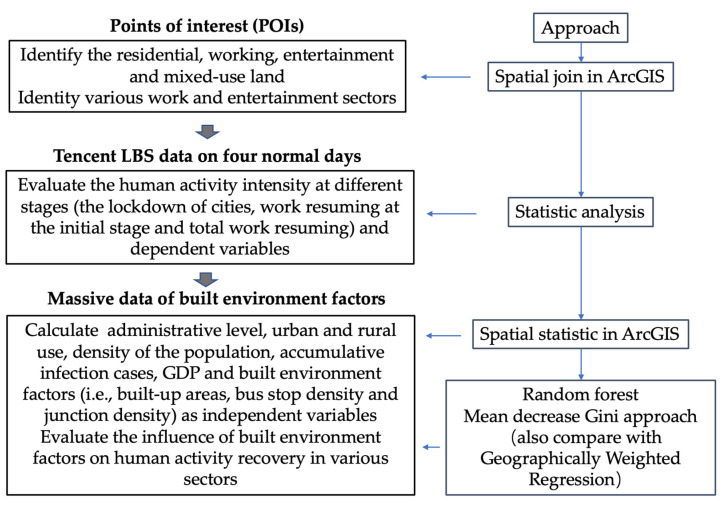
The flowchart of this study.

Table 3 illustrates a summary of the attributes and data sources used for the MDG approach at every grid cell. We chose the attributes from economic, demographic, administrative, built-environmental and epidemic perspectives. We clarify the study data and approach used in this study in a flowchart (Figure 2).

## 5. Results

In the following results, we firstly show the identified land-use category of each grid cell based on the method in Section 4.1, and then the human activity change is examined through the equations in Section 4.2. Furthermore, we reveal the influence of the grid-cell attributes on the change in human activity intensity through the MDG approach described in Section 4.3.

### 5.1. The Distribution of Land-Use Categories

**Figure 3:**
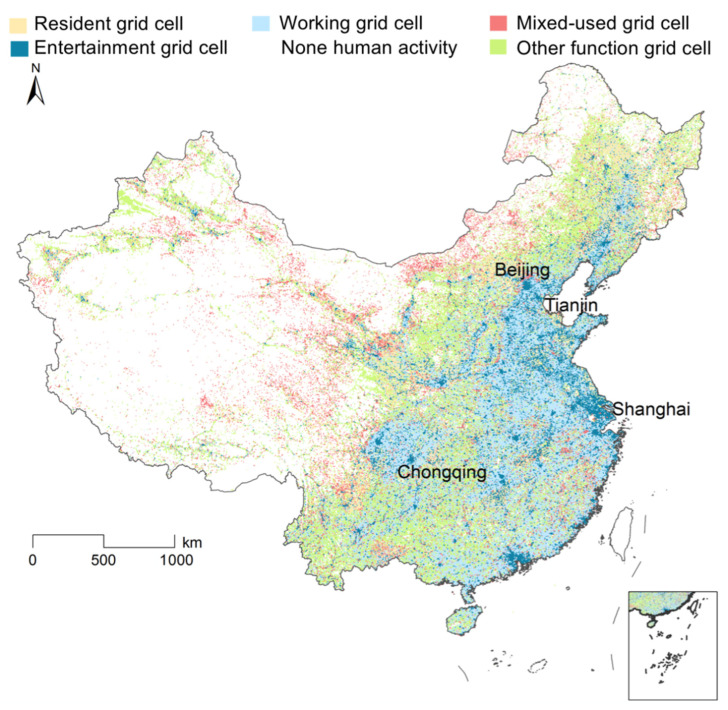
The spatial distribution of land use categories across China Mainland.

Figure 3 shows the spatial distribution of residential land, working land, entertainment land, mixed-use land and other function land across China. The working grid cells had a relatively large distribution in China, with a total of 65,085 grid cells. The residential grid cells (18,970 grid cells) and mixed-use pixels (21,123 grid cells) had a relatively similar amount. The number of entertainment grid cells was 14,226. The other function grid cell refers to parks, squares, transportation land, storage land, farmland and other land types and we did not consider this category in this study. The non-human activity areas refer to the wilderness, the mountain areas and the river areas.

### 5.2. Change Rate in Human Activity Intensity in the Three Periods

**Figure 4:**
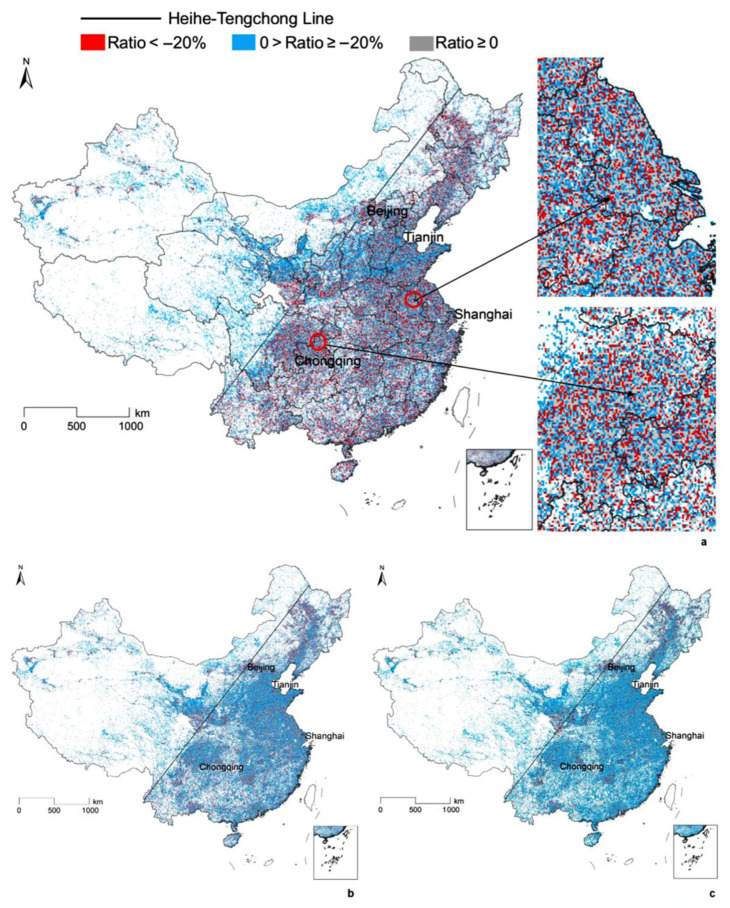
Human activity-intensity change rate in the three periods: (**a**) Change ratio in the first period, (**b**) Change ratio in the second period, (**c**) Change ratio in the third period.

The following figures (Figure 4a–c) illustrate the change rate in human activity intensity in the three periods studied: during the lockdown of cities, at the first stage of work resuming and at the total work-resuming stage. According to the figures, there is an obvious difference on the two sides of the Heihe–Tengchong line, which is an imaginary line that divides the area of China into two roughly equal parts. According to Naughton (2006), to the west of the line, 57% of the area has only 6% of the population, while the east of the line has 43% of the area, but 94% of the population [41]. The reduction rate on the eastern side of the Heihe–Tengchong line was higher than on the western side, with more grid cells having a reduction rate higher than −20% (in red).

There were 53,491 grid cells (occupying 44.80%) suffering from human activity intensity reduction among all of the residential, working, entertainment and mixed-use grid cells in the first period. For the total-work-resuming period, 52,433 grid cells, which occupied 43.91%, suffered from activity-intensity contraction. In addition, at the first stage, the average change in human activity intensity decreased by 25.02% during the lockdown when compared to normal conditions, while, for the total-work-resuming stage, the activity intensity decreased by only 6.51%. The average change in human activity decreased by 7.97%, which is close to the third period.

As shown in Table 4, the human activity intensity associated with commercial sites, sports/gyms and tourism experienced the relatively largest contraction (decreased by 34.76%, 33.20% and 31.10%, respectively) in China during the lockdown. Among the working sectors, the human activity intensities relating to private companies and education institutions had a relatively larger decrease, decreasing by 28.91% and 24.53%, respectively, during the lockdown.

At the first stage of work resuming, the average human activity intensity increased by 15.53% in the entertainment grid cells and 12.86% in the working grid cells when compared to the lockdown period. Commercial sites, sports/gyms and tourism more quickly resumed in human activity intensity with the increase percentages being 18.36%, 17.92% and 16.16%, respectively. Excluding education institutions, the human activity intensity associated with other working sectors had a similar average rise percentage after the lockdown. Government institutions, with a 13.89% rise in human activity intensity, were the highest among all the working sectors.

For total work resuming, the human activity intensity related to the commercial site, sport/gym and hotel sectors had relatively smaller contractions than the other entertainment types when compared to the normal conditions before the COVID-19 spread. In contrast, the human activity intensity decreased by 15.30% for catering sites, which is the highest percentage of all the work and entertainment sectors for total work resuming. In addition, the average reduction rate for private companies was the highest (−14.63%) among the other working sectors, with the rates being relatively smaller for government institutions and the legal sector.

### 5.3. The Influence of Grid-Cell Attributes on the Change in Human Activity Intensity

**Figure 5:**
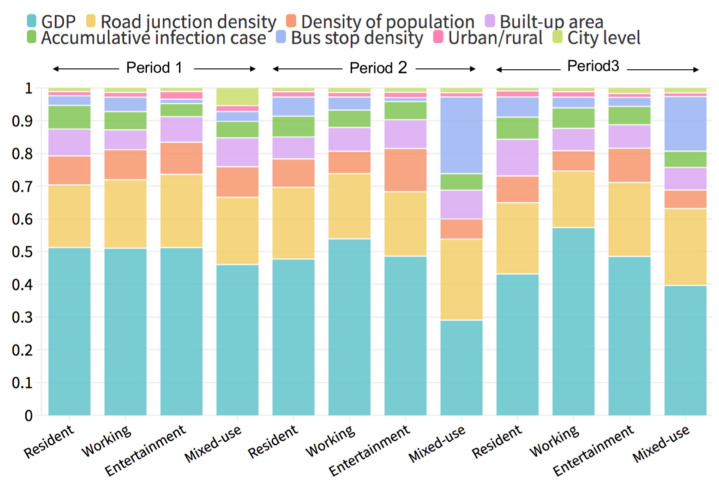
The importance of eight attributes on the change in human activity intensity in the different land-use categories in the three periods.

We further investigated the importance of eight attributes (see Table 3) on the change differences in the different periods. The attributes reflect the economy, demography, administration, built environment and epidemic information. As shown in Figure 5, the importance of the attributes to human activity change is relatively stable in each categorized land-use type in the different periods.

The GDP and road junction density had more of an influence on the human activity change in all the land-use categories. The importance of GDP occupied around 40–50% of the total importance in most land-use categories in all periods. Only for the mixed-used grid cells in the second period was the percentage lower than 40%, with a percentage of 29%, but GDP was still the influencing attribute for activity-intensity change. In addition, the percentage of importance for road junction density was around 20% in all periods in different land-use categories, and this attribute was especially important for the mixed-use grid cells (25%) in the second period, as well as the entertainment grid cells (23%) and working grid cells (23%) in the third period. 

The importance of density of population, built-up areas and cumulative infection cases were stable at a lower level and similar in the different land-use categories in different periods. The population density had a slightly greater influence on the entertainment grid cells in the second period. For the mixed-use grid cells in the second period and third period, bus stop density was also important. Furthermore, the administrative attributes, including the location (situated in urban or rural areas) and administrative level, were not important to change in human activity in relation to the efficiency of lockdown and relaxation policies.

Due to the GDP and road junctions being the two most important attributes causing the differences in human activity intensity, improving the equality of economic development and urban road infrastructure could reduce the differences in human activity-intensity change nationwide for each land-use type, in other words, improving the effectiveness of the lockdown and relation policies. In addition, the bus stop density was also an important factor causing differences in the human activity-intensity changes for mixed-use land. A relatively balanced distribution of bus stop density could also improve the policy effectiveness during a COVID-19 outbreak.

## 6. Discussion

It has been mentioned in the results that the eastern side of the Heihe–Tengchong line, which is more populated and economically developed, had a stronger human activity reduction rate. This is a very interesting phenomenon and may be caused by several aspects. One is the binary policy effectiveness in China, as argued by Ma (2017) [42]. The policy executors in the western area are less professional and knowledgeable when executing policies from the central government than those in the eastern area, due to the work opportunities in the eastern area being more competitive with higher salaries. Because of the higher political effectiveness, human activity intensities in the eastern area reduced to a large extent after the lockdown policy was issued by the central government. This is consistent with the study by Bonaccorsi et al. (2020), which relied on massive near-real-time Italian mobility and found that the impact on mobility during the lockdown was stronger in the municipalities with a higher fiscal capacity [26]. Furthermore, during relaxation, many provinces had no newly added infection cases in the western areas, while in the eastern areas with a large population and high human mobility some provinces still had newly confirmed infection cases; thus, human activity intensities were not quickly restored to the levels before the COVID-19 spread. The sense of civic duty, education level, economy development level, working arrangement, health concerns and tele-commuting coverage rate can also affect the different effects of lockdown and relaxation on the two sides of the Heihe–Tengchong line and we will further explore the reasons for this in detail.

Very few have investigated the human activity changes in different business sectors in China before. By using the VIIRS night-time light, Liu et al. (2020) suggested that human activity increased in the residential area and decreased in commercial centers for most of the provinces after the lockdown, whilst transportation and public facilities generally stayed the same [43]. However, the degree of human activity change was not evaluated in Liu et al.’s study. In this study, we suggest that commercial sites, sport/gyms and tourism among the entertainment sectors are the most impacted by lockdowns in China. We compared the human activity restriction with Italy, which was the first European country to apply a national lockdown in response to the spread of COVID-19. According to the COVID-19 community mobility report (2020), in Italy, the mobility trends associated with tourism, commercial and services also experienced a sudden contraction. More than 90% of the mobility in Italy was reduced during the lockdown, which is much higher than this study in China reflects. This may be caused by the relatively lower resolution of the Tencent LBS data. Each grid cell of the commercial sites, sport/gyms and tourism could also combine a few residential areas with certain human activity intensity increasing. Therefore, the real degree of reduction of human activity could be higher than that observed with the support of LBS data in China. According to previous studies, land use and social-economic factors are related to human activity recovery, for instance, the urban built environment with low density and high aging populations perform better in economic vitality recovery [44]. The compact development can decrease the population’s trips to shopping and entertainment places [45]. In this study, we also found that the population density was more important to entertainment sectors at the first and total recovery stages, while the road junction density played significant roles in human activity-intensity recovery at all stages. According to the result of the human activity-intensity change, this study proposes the following political suggestions. At the national level, we suggest that the government should provide more financial subsidies to private companies and encourage food delivery services for catering sectors during the COVID-19 outbreak, due to the human activity intensity decreasing significantly, even after the total work resuming. Policies to encourage eating outside, such as the “Go to eat” policy in Japan, could be proposed to promote restoration of these business sectors. In addition, policies to encourage tele-commuting work and to support the construction of 5 G facilities should also be implemented to maintain the business of private companies to a greater extent. Furthermore, as it was easier for human activity intensity to be restored with relaxation policies for the commercial site and sport/gym sectors than it was for other entertainment sectors, policies to encourage mask-wearing, maintain social distance and keep indoor air circulation with regard to these two sectors should be immediately implemented to avoid further waves of pandemic spread.

We also suggest spatial heterogeneous policies at two levels: the community level and the city level. Utilizing a study of human activity at grid cells of 5 km × 5 km is very difficult to guide COVID-19 policymaking without relying on the administrative boundaries. With the knowledge at the grid-cell level, we suggest conducting the policymaking based on the Chinese community, which is a basic administrative unit. For instance, in Beijing, there are 8357 communities with an average size of 3.87 km^2^. On average, seven to eight communities can cover a grid-cell size and could work together to control the COVID-19 spread in Beijing. Therefore, future cooperation amongst the different communities is necessary to tackle the COVID-19 spread in China. Detailed policy suggestions could be given after comparing the administrative boundary of communities and grid cells in the future and we will conduct further studies.

As the coronavirus is spreading globally, the authorities in different countries have implemented various policies to restrict human mobility, such as shutting down education institutions, limiting the work and mobility of people and restricting public transportation utilization for mobility restrictions. In Japan, policies such as “go to eat” and “go to camping” have also been proposed to encourage activity and reduce the huge economic costs of the COVID-19 pandemic. With the popularity of big data, which can make it easier to reflect human locations and mobility traces and capture human activity globally, we expect that our study will provide a method/perspective to bring benefits to detailed policy-making that can be optimized worldwide during the pandemic through combining the activity intensity of different business sectors.

## 7. Conclusions

Change in human activity intensity is a consequence of the complex combination of COVID-19 responses in China. Although the lockdown and relaxation policies of the central government are the same in scale and time in China, the human activity-intensity changes vary across the different land-use categories in different periods. Nevertheless, our study captured the change in human activity intensity through Tencent LBS data in three periods of the COVID-19 pandemic nationwide. We explored how the intensity changed and how the changes differed across the different work and entertainment sectors. We, accordingly, demonstrated the influences of land attributes to these differences.

The main findings of this study include the following: the human activity intensity reduction rate on the eastern side of the Heihe–Tengchong line was more serious than on the western side—human activity intensity on the eastern side decreased by 35%, 16% and 15%, respectively, compared to the normal conditions during the lockdown in the three periods, while the values were 22%, 9% and 6%, respectively, on the other side. It was easier for commercial sites and sport/gyms to be impacted by the lockdown and relaxation policies than the other entertainment sectors. The impact of COVID-19 on human activity intensity lasted longer for the catering sites, and even after work resuming, there was still a 15.30% intensity reduction. For the working sectors, the relaxation effect was greater in government institutions, but less effective in private companies. GDP, as well as road junction density were the two most important attributes causing the difference in human activity intensity. Bus stop density was also an important influence in human activity change in the first stage of work resuming for mixed-use land. The above findings are important for COVID-19 control and management in China at different stages.

Our findings shed light on the human activity-intensity consequences in the different stages of the COVID-19 pandemic. We suggest that the lockdown and relaxation seemed to have unequal impacts on the different sectors. This study has provided some practical contributions. First, during a lockdown, a financial subsidy could be provided to sectors where human activity has reduced too much between before and after the total work resuming. Second, spatial heterogeneous strategies should be implemented to guide human activity during the COVID-19 pandemic. Finally, a balanced development of the GDP degree and road network distance could be beneficial for reducing the difference in human activity-intensity changes nationwide to improve the effectiveness of lockdown and relative policies.

In closing, we acknowledge there are limitations in this study. Firstly, the LBS data has a low resolution. Therefore, to overcome this shortcoming in the future, data with a higher resolution should be used if possible. Secondly, identification of the different work and entertainment sectors may not be accurate through single POI data in the selected grid-cell scale. Thirdly, as well as the attributes we chose in this study, further attributes could be equally as important as the selected attributes. Further research could be conducted to extend this study. In this study we found that, in China, the reduction intensity rate was 30% in general; however, what is a suitable reduction rate still needs to be checked through epidemiology models in the next step in order to support more accurate policy-making.

## Figures and Tables

**Table 1:** The POIs categories to identify the land use categories.

Land Use Categories	First Level Categories
Residential land	Residential community
	Community service
Working land	Private company
	Office building
	Financial sector
	Legal sector
	Government institution
	Education institution
Entertainment land	Commercial site
	Hotel
	Tourism
	Sport/gym
	Catering site

**Table 2:** The comparison of the accuracy of models.

	MSE		Adjusted R^2^	
	GWR	RF	GWR	RF
Period 1_Residential	0.178	0.170	0.027	0.038
Period 1_Working	0.088	0.082	0.207	0.262
Period 1_Entertainment	0.043	0.040	0.534	0.546
Period 1_Mixed-Use	0.076	0.070	0.436	0.485
Period 2_Residential	0.276	0.275	0.022	0.029
Period 2_Working	0.041	0.039	0.454	0.511
Period 2_Entertainment	0.215	0.212	0.025	0.037
Period 2_Mixed-Use	0.095	0.093	0.037	0.061
Period 3_Residential	0.876	0.873	0.019	0.028
Period 3_Working	0.018	0.016	0.561	0.593
Period 3_Entertainment	0.087	0.085	0.270	0.371
Period 3_Mixed-Use	0.079	0.072	0.431	0.508

**Table 3:** Grid-cell attributes and data sources for the mean decrease Gini approach.

Perspective	Attribute	Data Source
Economy	GDP	Resource and Environment Science and Data Center in 2015
Demography	Density of population	Resource and Environmental Science Data Center of the Chinese Academy of Sciences in 2015
Administration	Urban/rural	Chinese administrative boundary in 2015
	Administrative level	China City Statistical yearbook 2015
Built environment	Built-up area	Resource and environment data cloud platform in 2015
	Bus stop density	Amap
	Junction density	Road network from open street map in 2020
Pandemic	Accumulative infection cases	Collected by the China University of Geoscience

**Table 4:** Change in the human activity intensity in the three periods for the entertainment and work sectors.

Type	Sub-Categories	First Period	Second Period	Third Period
Work	Private company	−28.91%	−15.85%	−14.63%
	Office building	−22.46%	−9.24%	−8.15%
	Financial sector	−23.52%	−10.06%	−8.14%
	Legal service	−23.72%	−10.76%	−7.52%
	Government institution	−23.79%	−9.90%	−6.45%
	Education institution	−24.53%	−13.94%	−8.25%
Entertainment	Commercial sites	−34.76%	−16.40%	−11.32%
	Hotel	−30.40%	−15.56%	−11.56%
	Tourism	−31.10%	−14.94%	−13.19%
	Sport/gym	−33.20%	−15.28%	−11.51%
	Catering site	−26.13%	−15.78%	−15.30%
